# Tracking Career Outcomes for Postdoctoral Scholars: A Call to Action

**DOI:** 10.1371/journal.pbio.1002458

**Published:** 2016-05-06

**Authors:** Elizabeth A. Silva, Christine Des Jarlais, Bill Lindstaedt, Erik Rotman, Elizabeth S. Watkins

**Affiliations:** 1 Office of Career and Professional Development, University of California, San Francisco, San Francisco, California, United States of America; 2 Office for Postdoctoral Scholars, University of California, San Francisco, San Francisco, California, United States of America; 3 Graduate Division, University of California, San Francisco, San Francisco, California, United States of America

## Abstract

The oversupply of postdoctoral scholars relative to available faculty positions has led to calls for better assessment of career outcomes. Here, we report the results of a study of postdoctoral outcomes at the University of California, San Francisco, and suggest that institutions have an obligation to determine where their postdoc alumni are employed and to share this information with current and future trainees. Further, we contend that local efforts will be more meaningful than a national survey, because of the great variability in training environment and the classification of postdoctoral scholars among institutions. We provide a framework and methodology that can be adopted by others, with the goal of developing a finely grained portrait of postdoctoral career outcomes across the United States.

## Abstract 2

This Perspective reports the results of a study of postdoctoral outcomes at UCSF and suggests that the same methodology could be used to help other institutions determine where their postdoc alumni are employed, with a view to sharing this information with current and future trainees.

## Introduction

Both the prodigious output and global preeminence of the United States academic research enterprise depend on the contributions of postdoctoral scholars (postdocs). We posit that postdocs participate in a system that evolved many decades ago, in which they receive mentoring intended to prepare them for independent academic research careers, in exchange for providing labor, producing data, writing manuscripts, and preparing grant applications. We believe this model disproportionately benefits those postdocs who move into faculty positions. Even in institutions with dedicated career professionals and services, the support and guidance for those who eventually move into non-faculty positions is a relatively small part of the complete postdoctoral experience, which is widely viewed as an apprenticeship for a faculty position. While recent literature suggests that only a minority of postdocs move into faculty positions, efforts to determine what postdocs do after their training, and to accurately determine the proportion in faculty positions, have consistently failed [1–3]. Over the past 20 years, at least three major reports [1–3], including the recently released *The Postdoctoral Experience Revisited* from the National Academy of Sciences, have called for improved tracking of the career outcomes of the nation’s postdocs as a prerequisite for addressing their training needs. These calls have largely gone unheeded.

Here, we report the results of our study of postdoctoral career outcomes at the University of California, San Francisco (UCSF)—which we believe to be the most comprehensive single-institution study conducted to date—and provide a framework and methodology that can be replicated at other institutions. This approach offers the transparency required for postdocs to make informed career decisions.

Our data reveal that outcomes vary according to training (PhD versus MD/PhD) and the location of employment (United States versus non-US). We discuss the false dichotomizing of faculty positions as “tenure-track” versus “non-tenure-track,” which fails to acknowledge the increasing nuance in academic appointments and misleads postdocs as they weigh their options. Finally, we argue that institutions cannot wait for change at the level of national policy; we propose that institutions must accept responsibility for providing better transparency of career outcomes to the fullest extent possible at a local level, to give postdocs timely and pertinent information to enable them to prepare for their futures.

## Postdoc Career Outcomes Study: Method and Findings

We focused our investigation on outcomes for PhDs who entered US-based employment from 2000 to 2013, compiled from the reports required under the Ruth L. Kirschstein National Research Service Award (T32) funding scheme of the National Institutes of Health (NIH) [4]. These reports list the current employment status and positions for all students and postdocs who trained in a lab participating in a T32 program, regardless of funding source (postdocs were supported by a variety of mechanisms, including direct T32 support, individual fellowship, faculty research grant, foreign support), and they require trainee follow-up for 10 years after leaving UCSF. Additional details of the T32 funding program are described in the supporting information.

**Fig 1:**
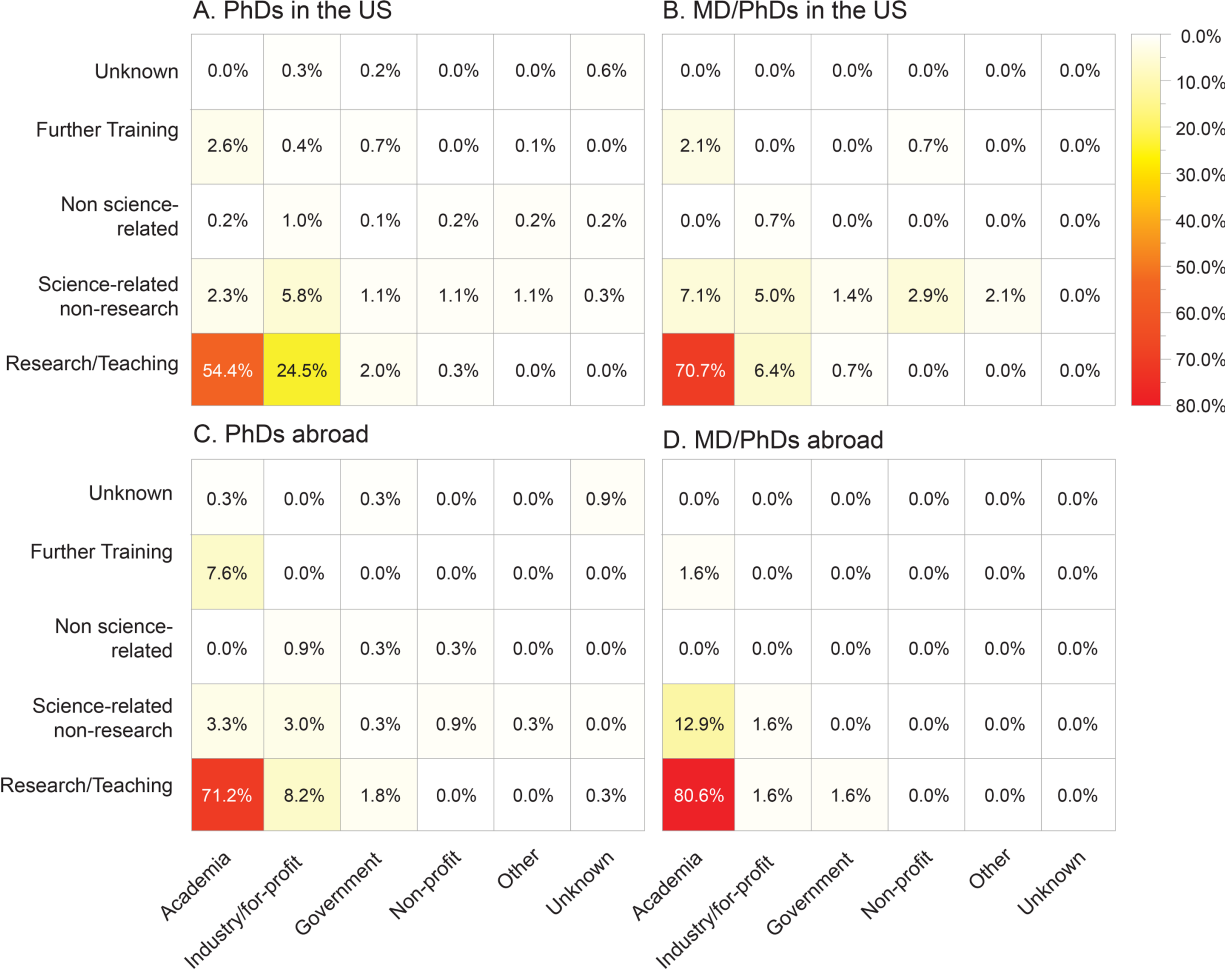
UCSF postdoc career outcomes organized by sector of the workforce, and by career-type, for (A) PhD-trained postdoc alumni employed in the US (*n* = 899), (B) MD/PhD-trained postdoc alumni employed in the US *(n* = 141), (C) PhD-trained postdoc alumni employed outside the US *(n* = 329), and (D) MD/PhD-trained postdoc alumni employed outside the US *(n* = 62).

When we commenced our study in the spring of 2014, UCSF had 55 active T32 awards. After excluding predoctoral students, clinical (non-research) fellows, trainees who were still at UCSF, and those who did not hold a PhD or PhD-equivalent, we were left with data for 1,431 postdoctoral scholars from 28 training programs (S1 Table), representing the research groups of 277 UCSF faculty. We estimate this figure to be 33% of all postdocs who separated from UCSF from 2000 to 2013 (see supplementary materials). We then conducted web searches to identify and confirm the job title and employer for each former trainee at the time of the study. For those in academic positions, we relied on PubMed, using the author information on published papers, and verified status using university websites. For those outside of academic research roles, we relied primarily on LinkedIn, corroborating our information using government, corporate, and non-profit organization websites (S1 Fig). We then classified job titles according to workforce sector and appointment category (Fig 1, S4 Table), which contrasts with other approaches undertaken in reports of graduate student outcomes and with our preliminary analysis of this dataset previously made available at http://postdocs.ucsf.edu/news/career-outcomes.

We initially investigated outcomes for PhDs without a medical degree (MD) who went into US-based employment (899 total). We wanted to better understand the career opportunities afforded by the extended and specialized training accrued specifically through postdoctoral research, to identify employers who choose to capitalize on this training, and to identify the sectors of the workforce in which these employers operate. We found 81% (731/899) of UCSF postdoc alumni were employed in areas typically thought of as PhD-related careers: research or teaching in academia and research in industry or government settings. If we include those working in science-related non-research positions (e.g., K–12 education, communication, policy, regulation, administration, and business development; S5 Table), the proportion engaged in the research enterprise climbs to 93% (837/899). Four percent (33/899) were getting further training, 2% (19/899) worked in non-science related jobs, and 1% (10/899) could not be accounted for.

**Fig 2:**
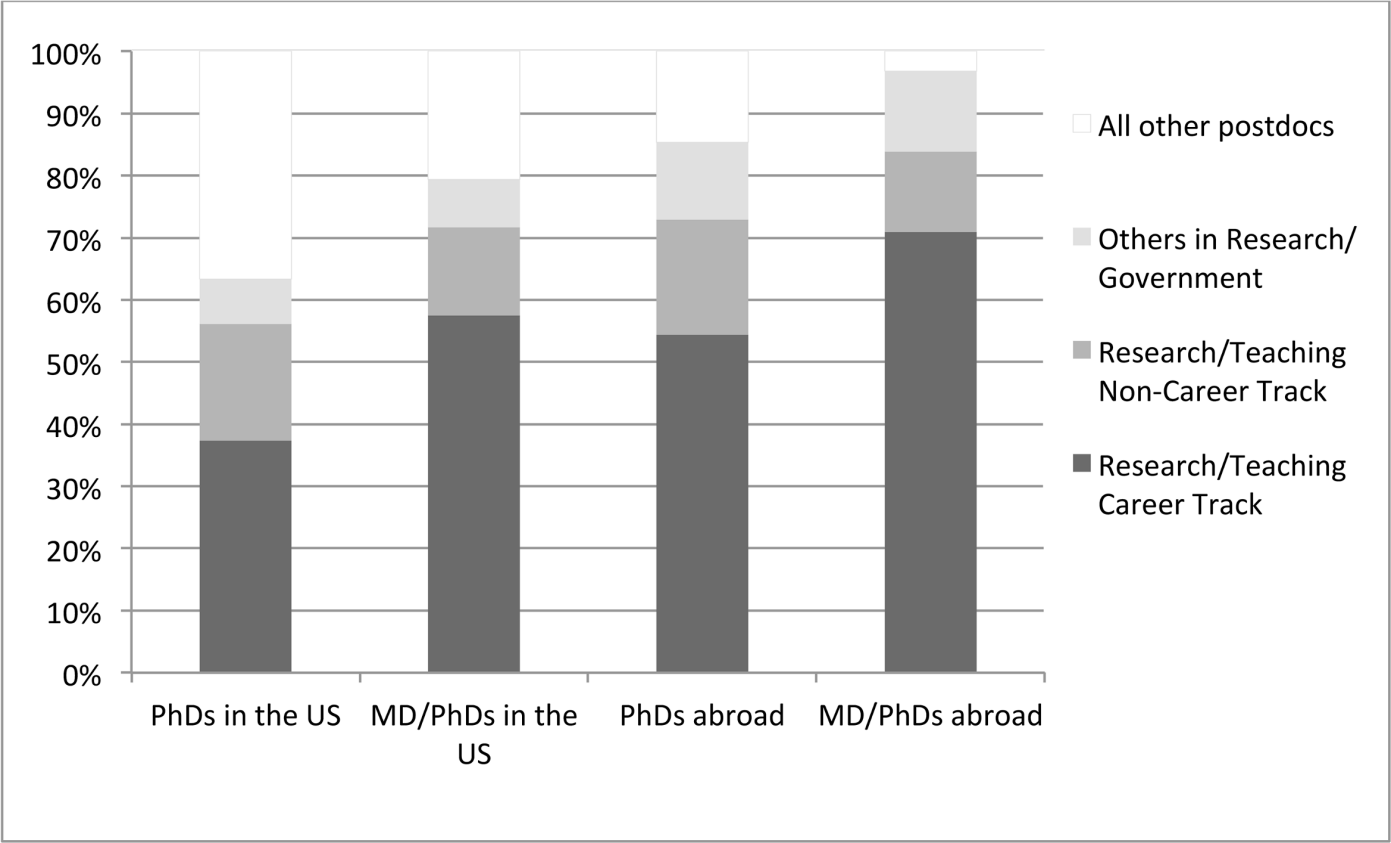
Postdoc alumni employed in academic and government positions, separated into three categories: those in faculty positions (i.e., with titles of assistant professor, associate professor, or full professor, or equivalent titles in other sectors, such as group leader or investigator); those in research and teaching positions that are not faculty; and those in positions that are not research or teaching. Figures are presented as a proportion of the full population of alumni included in this study.

Attainment of a faculty position in an academic setting is the most commonly cited primary career goal for postdoctoral trainees [5,6], so we next sought to determine the proportion of UCSF postdocs who had attained these positions. Our experience working with UCSF’s postdoctoral population through the Office of Career and Professional Development revealed that “desirable positions” also include appointments at government and non-profit research institutions, such as the NIH or the Gladstone Institutes. Thus, we asked what proportion of former UCSF postdocs attained academic faculty positions, including those holding titles of assistant, associate, or full professor at a university, or an equivalent title at a research or government institution (see Fig 2 and S4 Table). We found that 37% (336/899) of US-employed postdoc alumni were in faculty positions, that is, in faculty or faculty-like full-time research or teaching positions. We further classified the institutions at which these alumni were employed using a system based on the Carnegie classification framework (http://carnegieclassifications.iu.edu/) and determined that the majority of these faculty are employed at research institutions (see S6 Table).

Interestingly, we observed very different outcomes for two specific sub-populations of postdocs not included in the above calculations. First, of the 23% of UCSF’s postdoc alumni who had left the United States, 54% (179/329) were in faculty positions (Fig 2, S3 Table). The proportion of UCSF postdocs who are on non-resident visa status varies between 52% and 63% (S3 Table). Thus, less than half of international postdocs at UCSF leave the US following their training, and so this subgroup likely consists primarily of foreign nationals. It is unclear whether the differences we observed are due to differences in opportunities abroad or in the career aspirations of foreign nationals undertaking training at UCSF. Second, of the 10% of postdoc alumni employed in the US and who hold both a PhD and an MD (or equivalent), 57% (81/141) were in faculty positions (Fig 2). We believe that many of those MD/PhDs holding research faculty appointments are likely to have joint clinical appointments. Interestingly, the subgroup with the highest rate of placement in faculty positions consisted of MD/PhDs employed outside the US: 71% (44/62).

Faculty positions are often colloquially referred to as “tenure-track” positions, often featuring in career development resources and articles and in discussions of the biomedical workforce. However, we found that tenure-track is not an accurate description of many academic faculty appointments in the biomedical sciences at research universities: positions may be institution-funded or grant-funded (“hard money” or “soft money”) and may come with varying degrees of stability (either through a tenure commitment or other funding arrangement). Through our work at UCSF, we consistently observe that postdocs do not recognize that the term tenure-track is over-applied and used colloquially rather than as an accurate description of a given faculty appointment. The confusion is exacerbated by the absence of this distinction in public-facing profiles at most universities, leading postdocs to assume that a professor title is indicative of a tenure-line position. We wanted to better understand the proportion of faculty positions that might be considered truly tenure-line, so we looked at postdoctoral alumni employed as faculty at UCSF and determined their true status using data provided by UCSF’s Office of Academic Affairs. Of the UCSF postdoc alumni who attained UCSF faculty positions, 21% (19/92) are in the university’s tenure-track series.

To investigate the potential influence of individual training environments (the specific faculty mentor for each postdoc) on career outcomes, we calculated the percentage of postdoc alumni for every mentor with at least 10 postdocs in our dataset (see S7 Table). We observed considerable variation in the outcomes depending on the mentor (from a high of 93% to a low of 9% placement in faculty appointments), but we cannot draw conclusions about the training environment due to the small sample sizes. Institutions might consider encouraging faculty to publish their postdoc alumni career outcomes to provide greater transparency to prospective postdocs.

## What Are the Implications for Postdoctoral Scholars Nationwide?

We are aware that UCSF is not typical of US research institutions, in that our institution focuses almost exclusively on biomedical research and graduate-level training and receives the second largest amount of NIH funding (S3 Table) [7]. Thus, the career outcomes we present here will not be representative of a national average. A recent study revealed that 70%–85% of career-track positions in a diverse set of disciplines at ten different universities are filled by alumni from an elite 25% of institutions [8]. Inferring that faculty appointments in the biomedical sciences would be subject to the same trends, it is likely that UCSF postdocs move into faculty positions at a rate higher than the national average, and maybe far higher than some individual institutions. Yet just 37% of UCSF’s postdoctoral trainees secure faculty positions. Other major academic medical centers with comparable funding/research profiles may see results similar to ours, but we predict that postdocs employed outside the elite 25% of institutions are less likely to attain faculty positions.

We acknowledge that we cannot exclude the possibility of bias within our dataset. The postdocs in our study fall under a funding mechanism intended to improve training, which might be expected to affect career outcomes. However, these postdocs are in a range of labs, with mentors who may or may not have direct involvement with the grant, and are supported by a range of mechanisms. In addition, when we examine the career outcomes for postdocs according to individual labs and mentors, we see enormous variation in the proportion of alumni in faculty appointments (S7 Table), a result that is consistent with what might be expected from an analysis of the full postdoc alumni population.

National conversations frequently include discussion of the availability of “tenure-track” positions, because postdocs continue to cite tenure-track positions as their primary career aspiration. We propose that focusing on the number of tenure-track positions misleads postdocs in two ways: it fails to acknowledge the breadth of desirable independent research positions, and it oversimplifies the terms of employment under which principal investigators work. Academic faculty candidates negotiate increasingly nuanced and complex contracts that may not include tenure (e.g., 79% of our sample who are in UCSF faculty positions are non-tenure-track) and may not involve significant institutional commitment to salary. Knowing that job stability and institutional commitment may factor into many postdocs’ career decisions, we call for better transparency in the career outcomes of postdocs and the nature of available positions. This transparency should include more accurate application of the term “tenure-track.”

## Institutions Have a Responsibility to Take Action

We maintain that tracking career outcomes is the first step in addressing the career-planning needs of current and future postdocs. Building robust mechanisms for tracking postdoctoral populations as they move through their careers will enable detection of job trends and inform accompanying shifts in career support and training, and should also include demographic data that have not been historically available, such as gender, ethnicity, and age.

Importantly, new tracking efforts ought to be accompanied by a retrospective study. It would be easier for institutions to begin tracking career outcomes by surveying postdocs as they leave the institution, but it would then take years to reach a meaningful sample size, particularly at institutions with smaller postdoc populations. Such an effort will be of use to future postdocs and must be undertaken to meet their needs, but it fails those currently in the system. Equally, institutions should not rely on the minimal career outcomes reporting requirements for their T32 grants. Many institutions do not hold T32 training grants and therefore do not have reports to which they can refer. Where these reports do exist, they are often inadequate in that they are designed to be scanned quickly as one component of a lengthy grant application, not to be mined meticulously for data collection and analysis. The hard work must be done to corroborate the employment status of postdocs as reported in those grant application data tables.

Thus far, much of the conversation about postdocs and their roles in the scientific workforce has been enmeshed in wider discussions of biomedical research policy [9,10]. National initiatives are well intentioned, but the complexity of remodeling entrenched practices—and doing so in a way that will work for hundreds of different institutions—means that change will take many years. In addition, national data collection and aggregation will blur important distinctions among institutions of different sizes, sites, and emphases. We observed very different outcomes depending on academic training (medical degree or not) and employment location (US or non-US). Regional labor markets feature local concentrations of opportunities, even for PhD-level scientists [11,12], and recent work revealed that the specific field for a doctoral degree influences employment sector, job placement, and earning potential [12]. We predict that mapping of local trends will reveal distinct clusters of career outcomes, and assembling a collective portrait rather than a single aggregate dataset will be necessary to provide current and future postdocs with sufficient information to make informed career decisions.

In their frustration with the inertia of the system, many postdocs have resorted to grassroots actions [13]. We think that responsibility for action should not be delegated to postdocs themselves. Their grassroots efforts are highly commendable, but their success and sustainability depend on support from departments and institutions. With robust data on career outcomes in hand, institutions can readily deploy an abundance of existing and freely available information, resources, strategies, and curricula. The data can be used immediately as a career development resource, enabling current postdocs to become acquainted with the opportunities available to them. Coupling this information with the development of an alumni network will enable postdocs to connect more readily with opportunities for career exploration, identification of skills and knowledge gaps, and cultivation of professional networks. Importantly, sustainable programs can be implemented in a matter of weeks or months, not years, at relatively little expense.

Our analysis reveals that the overwhelming majority of UCSF postdoc alumni (93%) move into positions that meet institutional and national training mandates, as a highly skilled workforce supporting the biomedical research enterprise. Yet the implied agreement described above—research productivity in exchange for career mentoring toward a faculty position—may not address the true needs of the majority of postdocs. Our recommendations for action at the institutional level should not be viewed as an adequate substitute for wider commitment from funding agencies at the level of policy. Equally, individual institutions must not defer responsibility while waiting for a cultural shift to ripple through the entire biomedical research enterprise. In order for universities and research institutions to appropriately serve postdoctoral trainees while simultaneously acknowledging the valuable contributions they make to scientific discovery, we advocate deployment of the methodology described here. Meaningful and long-lasting change must begin at the level of the institution.

## Supporting Information

S1 Data Anonymized data analyzed in this study. (XLSX)

S1 Fig Workflow for finding and verifying employment status for UCSF postdoc alumni. (EPS)

S2 Fig Career outcome according to duration of postdoctoral appointment at UCSF. (EPS)

S1 Table Ruth L. Kirschstein National Research Service Awards (T32) included in this study. (DOCX)

S2 Table All postdoc alumni included in this study, by employment location and degree type. (DOCX)

S3 Table UCSF postdoctoral scholar population by citizenship and gender. (DOCX)

S4 Table UCSF postdoctoral scholar population by age. (DOCX)

S5 Table Categories by sector and job type. (DOCX)

S6 Table Breakdown of the types of academic institutions in which career-track UCSF postdoc alumni are employed *(n* = 417). (DOCX)

S7 Table Faculty outcomes according to individual mentor/lab. (DOCX)

S1 Text Supporting information, including methodology. (DOCX)
