# Correction: Tracking Career Outcomes for Postdoctoral Scholars: A Call to Action

**DOI:** 10.1371/journal.pbio.1002600

**Published:** 2017-02-23

**Authors:** Elizabeth A. Silva, Christine Des Jarlais, Bill Lindstaedt, Erik Rotman, Elizabeth S. Watkins

The Funding section is incorrect. The correct funding information is as follows: EAS and BL received salary support from NIH grant 1DP7OD018420-01. This work is unrelated to grant activities. The funder had no role in study design, data collection and analysis, decision to publish, or preparation of the manuscript.
