# Supplementary material for: Tracking Career Outcomes for Postdoctoral Scholars: A Call to Action
Source: PLoS Biol. 2016 May 6;14(5):e1002458. doi: 10.1371/journal.pbio.1002458 (PMC4859534; doi:10.1371/journal.pbio.1002458)
Supplement: S1 Table — (DOCX) [file pbio.1002458.s004.docx]

**S1 Table.** Ruth L. Kirschstein National Research Service Awards (T32) included in this study.

| **Principle Investigator** | **Title of Grant** | **School: Department** | **Funding Agency** |
| --- | --- | --- | --- |
| Adler, Nancy E. | Psychology and Medicine: An Integrative Research Approach | SOM:Psychiatry | NIMH |
| Arean, Patricia A. | Clinical Services Research Training Program | SOM:Psychiatry | NIMH |
| Burchard, Esteban G. | Clinical Pharmacology, Drug Action & Pharmacogenetics | SOP:Pharmacology | NIGMS |
| Chambers, Henry | Biology of Infectious Disease Training Program | SOM:Medicine | NIAID |
| Copenhagen, David | Training Program for the Visual Sciences | SOM:Physiology | NEI |
| Coughlin, Shaun R. | Molecular and Cellular Basis of Cardiovascular Disease | SOM:Cardiovascular Research Institute | NHLBI |
| DeFranco, Anthony | Molecular and Cellular Immunology | SOM:Microbiology & Immunology | NIAID |
| DenBesten, Pamela | Comprehensive Oral Health Training Program | SOD:Orofacial Sciences | NIDCR |
| Engel, Joanne N. | Microbial Pathogenesis and Host Defense | SOM:Microbiology & Immunology | NIAID |
| German, Michael S. | Diabetes, Endocrinology & Metabolism Training Program | SOM:Medicine | NIDDK |
| Havlir, Diane | Training in HIV Translational Research | SOM:Medicine | NIAID |
| Hecht, Frederick M. | Training Researchers in Clinical Integrative Medicine (TRIM) | SOM:Medicine | NCCAM |
| Hellman, Judith | Comprehensive Anesthesia Research Training | SOM:Anesthesia & Perioperative Care | NIGMS |
| Heyman, Melvin B. | Training Program in Pediatric Gastroenterology/Nutrition | SOM:Pediatrics | NIDDK |
| Kegeles, Susan M. | Traineeships in AIDS Prevention Studies | SOM:Medicine | NIMH |
| Lee, Kathryn A. | Nursing Research Training in Symptom Management | SON:Family Health Care Nursing | NINR |
| Lovett, David | Academic Training Program in Nephrology | SOM:Medicine | NIDDK |
| Ma, Averil | INRSA Training Grant in Gastroenterology | SOM:Medicine | NIDDK |
| Maher, Jacquelyn J. | NRSA Hepatology Training Grant | SOM:Medicine | NIDDK |
| Mellon, Synthia | Integrated Training in Reproductive Sciences | SOM:Obstetrics, Gynecology & Rep Sci | NICHD |
| Miller, Walter L. | Training Grant in Pediatric Endocrinology | SOM:Pediatrics | NIDDK |
| Portillo, Carmen J. | HIV/AIDS Nursing Care and Prevention | SON:Community Health Systems | NINR |
| Shannon, Kevin | Research Training in Childhood Cancer | SOM:Pediatrics | NCI |
| Sheppard, Dean | Multidisciplinary Training Program in Lung Disease | SOM:Medicine | NHLBI |
| Slavotinek, Anne M. | Postdoctoral Training in Medical Genetics | SOM:Pediatrics | NIGMS |
| Sorensen, James L. | Drug Abuse Treatment/Services Research Training Program | SOM:Psychiatry | NIDA |
| Srivastava, Deepak | Training in Development Cardiovascular Biology | SOM:Pediatrics | NHLBI |
| Werb, Zena | Molecular and Cellular Mechanisms in Cancer | SOM:Anatomy | NCI |
