# Supplementary material for: Tracking Career Outcomes for Postdoctoral Scholars: A Call to Action
Source: PLoS Biol. 2016 May 6;14(5):e1002458. doi: 10.1371/journal.pbio.1002458 (PMC4859534; doi:10.1371/journal.pbio.1002458)
Supplement: S2 Table — (DOCX) [file pbio.1002458.s005.docx]

**S2 Table. All postdoc alumni included in this study, by employment location and degree type**

| Employed abroad | 27% | 391 |
| --- | --- | --- |
| Employed in the US | 73% | 1040 |
| PhDs | 86% | 1228 |
| MD/PhDs | 14% | 203 |
| **Total** | **100%** | **1431** |
