# Supplementary material for: Tracking Career Outcomes for Postdoctoral Scholars: A Call to Action
Source: PLoS Biol. 2016 May 6;14(5):e1002458. doi: 10.1371/journal.pbio.1002458 (PMC4859534; doi:10.1371/journal.pbio.1002458)
Supplement: S3 Table — (DOCX) [file pbio.1002458.s006.docx]

**S3 Table. UCSF Postdoctoral scholar population by citizenship and gender**

| **Year** | **Females** | | **Males** | | **US citizens and Permanent residents** | | **Internationals** | | **Total**** |
| --- | --- | --- | --- | --- | --- | --- | --- | --- | --- |
| 2005 | 45% | 472 | 55% | 575 | 37% | 389* | 63% | 658* | **1047** |
| 2006 | 45% | 491 | 55% | 592 | 45% | 491* | 55% | 592* | **1083** |
| 2007 | 44% | 455 | 56% | 588 | 39% | 406* | 61% | 637* | **1043** |
| 2008 | 45% | 473 | 55% | 575 | 44% | 465 | 56% | 583 | **1048** |
| 2009 | 46% | 484 | 54% | 572 | 46% | 482 | 54% | 574 | **1056** |
| 2010 | 48% | 526 | 52% | 576 | 47% | 518 | 53% | 584 | **1102** |
| 2011 | 48% | 516 | 52% | 569 | 46% | 502 | 54% | 583 | **1085** |
| 2012 | 48% | 515 | 52% | 555 | 48% | 510 | 52% | 560 | **1070** |
| 2013 | 49% | 530 | 51% | 558 | 48% | 518 | 52% | 569 | **1087** |
| 2014 | 51% | 542 | 49% | 519 | 48% | 508 | 52% | 553 | **1061** |
| 2015 | 53% | 563 | 47% | 503 | 46% | 496 | 54% | 573 | **1069** |
| *permanent residents are classified as "international" | | | | |  |  |  |  |  |
| **Total taken on December 1 in a given year | | | | | |  |  |  |  |
