# Supplementary material for: Tracking Career Outcomes for Postdoctoral Scholars: A Call to Action
Source: PLoS Biol. 2016 May 6;14(5):e1002458. doi: 10.1371/journal.pbio.1002458 (PMC4859534; doi:10.1371/journal.pbio.1002458)
Supplement: S4 Table — (DOCX) [file pbio.1002458.s007.docx]

**S4 Table. UCSF Postdoctoral scholar population by age**

| Year | <30 | 30-34 | 35-39 | 40-44 | 45-49 | 50+ | **Total**** |
| --- | --- | --- | --- | --- | --- | --- | --- |
| 2005 | 169 | 552 | 239 | 65 | 12 | 10 | 1047 |
| 2006 | 196 | 544 | 259 | 56 | 13 | 15 | 1083 |
| 2007 | 175 | 562 | 246 | 42 | 9 | 9 | 1043 |
| 2008 | 166 | 600 | 228 | 31 | 13 | 10 | 1048 |
| 2009 | 161 | 617 | 216 | 45 | 10 | 7 | 1056 |
| 2010 | 172 | 617 | 261 | 34 | 11 | 7 | 1102 |
| 2011 | 173 | 614 | 248 | 33 | 11 | 6 | 1085 |
| 2012 | 177 | 613 | 239 | 30 | 6 | 5 | 1070 |
| 2013 | 178 | 617 | 244 | 42 | 3 | 3 | 1087 |
| 2014 | 161 | 634 | 224 | 36 | 4 | 2 | 1061 |
| 2015 | 146 | 624 | 235 | 45 | 9 | 2 | 1061 |

**Total taken on December 1 in a given year
