# Supplementary material for: Tracking Career Outcomes for Postdoctoral Scholars: A Call to Action
Source: PLoS Biol. 2016 May 6;14(5):e1002458. doi: 10.1371/journal.pbio.1002458 (PMC4859534; doi:10.1371/journal.pbio.1002458)
Supplement: S5 Table — (DOCX) [file pbio.1002458.s008.docx]

**S5 Table. Categories by sector and job type**

| **Sectors** |  |
| --- | --- |
| Academic | Universities, colleges, and research institutions. Also included in this category are hospitals and non-profit research institutions (e.g., the Gladstone Research Institutes), owing to the similarity in the type of research conducted, the roles and responsibilities of the faculty, and the difficulty in disambiguating university hospitals from other hospitals. |
| Government | Municipal, county, state, federal government |
| Industry/for-profit | For-profit company |
| Non-profit | Non-profit organizations, excluding research institutes and hospitals |
| Other | Unemployed, self-employed, or in private practice |
| Unknown | Trainee outcome is unknown, or the type of organization could not be determined |
| **Career Types** |  |
| Research/Teaching | Conducting research or directing a program of research, those teaching in higher education, and those in a combination of these roles. |
| Science-related non-research | Work in a science-related job or area, but who do not conduct research. Examples are physicians, patent attorneys in the sciences, science communicators, K-12 education, and those working in policy, consulting, and university administrative positions. |
| Non-science | In a position unrelated to science research or education |
| Further training | In a subsequent postdoc, or undertaking further study |
| Unknown | Trainee outcome is unknown, or the job type cannot be determined |
| **Career-track vs. non career-track** | |
| Career-track | Academics with titles of assistant, associate or full professors, or equivalent titles in other sectors, such as group leader or investigator |
| Non career-track | Staff scientists, and academics with a professor title that included a qualifier such as adjunct or clinical |
