# Supplementary material for: Tracking Career Outcomes for Postdoctoral Scholars: A Call to Action
Source: PLoS Biol. 2016 May 6;14(5):e1002458. doi: 10.1371/journal.pbio.1002458 (PMC4859534; doi:10.1371/journal.pbio.1002458)
Supplement: S6 Table — (DOCX) [file pbio.1002458.s009.docx]

**S6 Table. Breakdown of the types of academic institutions in which career-track UCSF postdoc alumni are employed (N= 417).**

| **Institution Type** | **Research Institution** | **Master’s College** | **Baccalaureate College** | **Community College** |
| --- | --- | --- | --- | --- |
| Proportion of postdocs | 88% | 8% | 3% | 1% |
