# Supplementary material for: Tracking Career Outcomes for Postdoctoral Scholars: A Call to Action
Source: PLoS Biol. 2016 May 6;14(5):e1002458. doi: 10.1371/journal.pbio.1002458 (PMC4859534; doi:10.1371/journal.pbio.1002458)
Supplement: S7 Table — (DOCX) [file pbio.1002458.s010.docx]

**S7 Table. Faculty outcomes according to individual mentor/lab**

| Mentor ID* | Number of postdocs in this study | Number in faculty appointments | Percent in faculty appointments |
| --- | --- | --- | --- |
| 21 | 14 | 13 | 93% |
| 48 | 10 | 9 | 90% |
| 26 | 14 | 12 | 86% |
| 41 | 10 | 7 | 70% |
| 46 | 10 | 7 | 70% |
| 28 | 13 | 9 | 69% |
| 12 | 16 | 11 | 69% |
| 19 | 15 | 10 | 67% |
| 33 | 12 | 8 | 67% |
| 25 | 14 | 9 | 64% |
| 39 | 11 | 7 | 64% |
| 6 | 21 | 13 | 62% |
| 13 | 16 | 9 | 56% |
| 34 | 11 | 6 | 55% |
| 38 | 11 | 6 | 55% |
| 29 | 13 | 7 | 54% |
| 2 | 30 | 16 | 53% |
| 47 | 10 | 5 | 50% |
| 3 | 27 | 13 | 48% |
| 11 | 17 | 8 | 47% |
| 17 | 15 | 7 | 47% |
| 1 | 35 | 16 | 46% |
| 15 | 16 | 7 | 44% |
| 16 | 16 | 7 | 44% |
| 5 | 23 | 10 | 43% |
| 32 | 12 | 5 | 42% |
| 44 | 10 | 4 | 40% |
| 7 | 20 | 8 | 40% |
| 9 | 18 | 7 | 39% |
| 37 | 11 | 4 | 36% |
| 20 | 15 | 5 | 33% |
| 49 | 10 | 3 | 30% |
| 23 | 14 | 4 | 29% |
| 24 | 14 | 4 | 29% |
| 8 | 18 | 5 | 28% |
| 36 | 11 | 3 | 27% |
| 18 | 15 | 4 | 27% |
| 14 | 16 | 4 | 25% |
| 10 | 17 | 4 | 24% |
| 27 | 13 | 3 | 23% |
| 42 | 10 | 2 | 20% |
| 43 | 10 | 2 | 20% |
| 45 | 10 | 2 | 20% |
| 4 | 26 | 5 | 19% |
| 31 | 12 | 2 | 17% |
| 30 | 13 | 2 | 15% |
| 22 | 14 | 2 | 14% |
| 40 | 10 | 1 | 10% |
| 35 | 11 | 1 | 9% |

*Each mentor in our dataset is represented by a unique mentor ID
