# Supplementary material for: Tracking Career Outcomes for Postdoctoral Scholars: A Call to Action
Source: PLoS Biol. 2016 May 6;14(5):e1002458. doi: 10.1371/journal.pbio.1002458 (PMC4859534; doi:10.1371/journal.pbio.1002458)
Supplement: S1 Text — (DOCX) [file pbio.1002458.s011.docx]

## Supporting Information

**Tracking Career Outcomes for Postdoctoral Scholars: A Call to Action**

Elizabeth A. Silva,^1^ Christine Des Jarlais,^2,3^ William Lindstaedt,^1,2^ Erik Rotman,^2,3^ and Elizabeth S. Watkins.^3^

^1^Office of Career and Professional Development, ^2^Office for Postdoctoral Scholars, and ^3^Graduate Division, University of California San Francisco (UCSF), San Francisco CA 94158

**Detailed methods**

*Primary data sources*

We began our study in January 2014, gathering postdoctoral career outcomes data using the reports assembled for the Ruth L. Kirschstein National Research Service Award (T32) funding program. Under this mechanism, a single principle investigator applies for the grant on behalf of a department or group of labs, to secure a centralized source of graduate student and postdoctoral scholar training funds. A single research group may fall under the umbrella of more than one T32 award, and each award may support trainees in multiple labs. Principal investigators for each award are required to report career title information for a 10-year period for all postdocs in all research groups participating in the T32 program, regardless of the funding source supporting the postdoc’s salary.

At the time of study, UCSF held a total of 55 T32 awards. We first excluded those programs that were exclusively for graduate students, exclusively for medical doctors, or specifically clinical in nature. The remaining 36 awards (65%) include predoctoral PhD students and postdoctoral scholars who hold PhDs and/or MDs. We contacted the principle investigators (PIs) for each of these 36 programs to request copies of Tables 5B and 12B from their competitive renewals, for trainees with appointments between 2000 and 2013. Twenty-eight PIs provided the requested data, three programs were too new to include useful information, and five did not respond after numerous attempts to contact them by phone and email. Data from the remaining 28 programs are included here. The titles, departments, and PIs of each T32 award included in this study are listed in S1 Table. Note that these programs cover trainees from the research groups of a total of 277 individual faculty members.

Tables 5B and 12B were provided as PDF and/or Word documents (<http://grants.nih.gov/grants/funding/424/datatables_sampleall.pdf>). These were converted to Excel and combined into a single spreadsheet. We then excluded graduate students, current postdocs, postdocs who trained at an institution other than UCSF, and all trainees who did not hold a PhD or PhD equivalent (e.g., DDS). We also excluded clinical fellows. A clinical fellow was defined as a trainee seeking advanced medical training, some or all of which is undertaken in a clinical setting. Postdocs included in our study were those who devoted 100% of their time to research activities. In some cases, this activity was discerned by the degrees held: a postdoc holding a PhD without an MD was classified as a researcher. Where a postdoc held an MD, classification was inferred from the PI’s department and role at UCSF, and from the title of their research project. We then identified and eliminated duplicate entries deriving from the reporting of a single trainee on multiple T32 awards. Status as a duplicate entry was verified by comparing the name, former academic institution, degrees and dates received, topic studied, and career outcome.

After removing duplicates and excluding individuals not relevant to the scope of this study, we were initially left with 1,719 postdocs; subsequent refinement of the data winnowed that number to 1,431 unique individuals (S2 Table). UCSF does not have a record of the total number of postdocs who left during this period, so to estimate the proportion of UCSF postdocs accounted for in our study, we turned to datasets representing the annual average postdoc population. UCSF has annually employed between 1,000 and 1,200 postdoctoral scholars in basic biological, biomedical, and behavioral sciences for at least the past 15 years (S3, S4 Tables). According to UCSF Human Resources data, an average of 313 postdocs separate from the university each year. Assuming 313 separations per year for 14 years, we inferred a total of 4,382 left UCSF between 2000 and 2013. Thus our sample represents approximately 33% of UCSF’s postdoc population.

*Verifying/Updating Career Outcomes*

We verified or updated career outcomes through web searches. Not all of the career outcomes reports were current or accurate, so we confirmed status through use of multiple search criteria (name, degree/institution/year received, UCSF training dates, PI affiliation, research topic, and career outcome) and multiple sources. For those in academic positions, we relied on PubMed, using the author information on published papers and university websites. For those in other positions, we relied primarily on LinkedIn, corroborating our information using government, corporate, and non-profit organization websites (S1 Fig).

Where a career outcome could not be found or verified the trainee was categorized as “unknown.” Reasons for an “unknown” result included: a common name for which we could not uncover a full name or verify a match; inability to find an online presence beyond their affiliation with UCSF as a postdoc; and inability to identify current information even though previous employment was known. Deceased individuals were categorized according to their last known career position. Where a postdoc was indicated as having more than one career outcome in two sectors at the same time, we categorized for both (e.g., a career in academia and government research). These categories constitute less than 1% of our sample and are included in Fig 1.

*Categorizing career outcomes*

Trainees were separately categorized according to sector of the workforce (employer) and career type (job title), resulting in 30 distinct categories. An earlier dataset released at <http://postdocs.ucsf.edu/news/career-outcomes> relied on the six categories used in the 2012 NIH Biomedical Workforce Report (*6*). The newer framework we have described here is compatible with publicly available reports but is more flexible, allowing for additional analyses as desired. Those in research/teaching positions in academia or government were further classified into career-track and non-career-track. See S5 Table for detailed definitions.

*Categorizing of institutions according to the Carnegie Framework*

We used the Carnegie Classification framework [http://carnegieclassifications.iu.edu/descriptions/basic.php] to categorize the academic and government institutions at which UCSF postdoc alumni are employed as career-track faculty (S6 Table). Research Institutions include government research labs such as NIH or DOE labs, private research institutes such as the Gladstone Institutes or the Buck Institute for Research on Aging, and universities classified within the Carnegie system as RU/VH Research Universities (very high research activity) and RU/H Research Universities (high research activity). Master’s

Colleges include institutions listed in the Carnegie system as DRU Doctoral/Research Universities, or under any of the Master’s Colleges and Universities designations. Baccalaureate Colleges include those institutions listed under any of the Baccalaureate College designations. Associate’s Colleges include those institutions listed under any of the Associate’s College designations.

*Variation in career outcomes as a function of research mentor/lab and length of postdoctoral appointment*

We examined the variation in the proportions of faculty vs. non-faculty appointments according to both the individual training environment (the specific faculty mentor for each postdoc) and duration of the postdoctoral appointment at UCSF. Only those PIs/mentors with at least 10 postdocs in our dataset are listed here. To examine the relationship between career outcome and duration of the postdoctoral appointment, we subtracted the start year from the end year for each postdoc. We observe that postdoc alumni move into faculty appointments as soon as one year following their initial appointment at UCSF (S2 Fig), however it should be noted that these trainees may be in their second or third postdoc, a variable that is not tracked in our dataset. We also observe that a lengthy postdoc is likely in preparation for a faculty outcome (7 years or more).
